# Collecting eco‐evolutionary data in the dark: Impediments to subterranean research and how to overcome them

**DOI:** 10.1002/ece3.7556

**Published:** 2021-05-01

**Authors:** Stefano Mammola, Enrico Lunghi, Helena Bilandžija, Pedro Cardoso, Volker Grimm, Susanne I. Schmidt, Thomas Hesselberg, Alejandro Martínez

**Affiliations:** ^1^ Laboratory for Integrative Biodiversity Research (LIBRe) Finnish Museum of Natural History (LUOMUS) University of Helsinki Helsinki Finland; ^2^ Dark‐MEG: Molecular Ecology Group Water Research Institute (IRSA) National Research Council (CNR) Verbania Italy; ^3^ Key Laboratory of the Zoological Systematics and Evolution Institute of Zoology Chinese Academy of Sciences Beijing China; ^4^ Museo di Storia Naturale dell'Università degli Studi di Firenze “La Specola” Firenze Italy; ^5^ Department of Molecular Biology Rudjer Boskovic Institute Zagreb Croatia; ^6^ Department of Ecological Modelling Helmholtz Centre for Environmental Research – UFZ Leipzig Germany; ^7^ Plant Ecology and Nature Conservation University of Potsdam Potsdam Germany; ^8^ German Centre for Integrative Biodiversity Research (iDiv) Halle‐Jena‐Leipzig Leipzig Germany; ^9^ Institute of Hydrobiology Biology Centre CAS České Budějovice Czech Republic; ^10^ Department of Zoology University of Oxford Oxford UK

**Keywords:** anchialine, *Asellus aquaticus*, *Astyanax*, cave laboratory, computer simulations, experimental design, groundwater, model system, natural laboratory, nonmodel organisms, sampling strategy, stygobite, troglobite

## Abstract

1. Caves and other subterranean habitats fulfill the requirements of experimental model systems to address general questions in ecology and evolution. Yet, the harsh working conditions of these environments and the uniqueness of the subterranean organisms have challenged most attempts to pursuit standardized research.
2. Two main obstacles have synergistically hampered previous attempts. First, there is a *habitat impediment* related to the objective difficulties of exploring subterranean habitats and our inability to access the network of fissures that represents the elective habitat for the so‐called “cave species.” Second, there is a *biological impediment* illustrated by the rarity of most subterranean species and their low physiological tolerance, often limiting sample size and complicating laboratory experiments.
3. We explore the advantages and disadvantages of four general experimental setups (*in situ*, *quasi in situ*, *ex situ*, and *in silico*) in the light of habitat and biological impediments. We also discuss the potential of indirect approaches to research. Furthermore, using bibliometric data, we provide a quantitative overview of the model organisms that scientists have exploited in the study of subterranean life.
4. Our over‐arching goal is to promote caves as model systems where one can perform standardized scientific research. This is important not only to achieve an in‐depth understanding of the functioning of subterranean ecosystems but also to fully exploit their long‐discussed potential in addressing general scientific questions with implications beyond the boundaries of this discipline.

## Graphical abstract

We discuss best practices and novel ideas for performing standardized research in subterranean ecosystems, by focusing on key impediments, experimental ideas, and model systems.

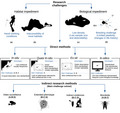

## 1 INTRODUCTION

For a *Homo sapiens*—a clumsy vertebrate inhabiting a primarily lighted world—to enter a cave is enterprising. As the sunlight fades, the air becomes moist, and a maze of passages opens in front of us, our first instinct as humans is to dismiss the subsurface world as one of the most inhospitable environments on Earth. Mentions to this apparent extremeness emerge in most caving stories (MacNeil & Brcic, 2017) insofar as speleology is indeed physically demanding and potentially hazardous (Zagmajster et al., 2010). However, by over‐emphasizing this anthropocentric view of caves, we tend to dismiss a different reality: Caves are not so extreme from the perspective of the eyeless and depigmented organisms that have adapted to living in darkness, which in contrast experience the exposure to sunlight and the wide climatic fluctuation of the outside world as harmful threats (Mammola, 2020). Interestingly, this dichotomous interpretation has framed the two main approaches followed by researchers over recent years: Those who have studied subterranean habitats as unique entities versus those who have used them as model to answer general scientific questions beyond the boundaries of subterranean biology (Martínez & Mammola, 2020).

Scientists across several generations have been aware of the potential of subterranean ecosystems (Box 1) as eco‐evolutionary models (Poulson & White, 1969), developing innovative methodologies and creative experimental designs to face the challenges associated with subterranean exploration. Thanks to these efforts, we have been able to tackle important subjects in ecology (Mammola, 2019), ethology (Parzefall, 1982), and evolution (Juan et al., 2010), ultimately reaching conclusions relevant to disciplines as diverse as medicine (Riddle et al., 2018; Stockdale et al., 2018; Yoshizawa et al., 2018), engineering (Lepore et al., 2012), and exobiology (Northup et al., 2011). Under this perspective, and despite the numerous obstacles to research, subterranean habitats may well qualify as frontiers for modern scientific research (Mammola et al., 2020).

**FIGURE 1:**
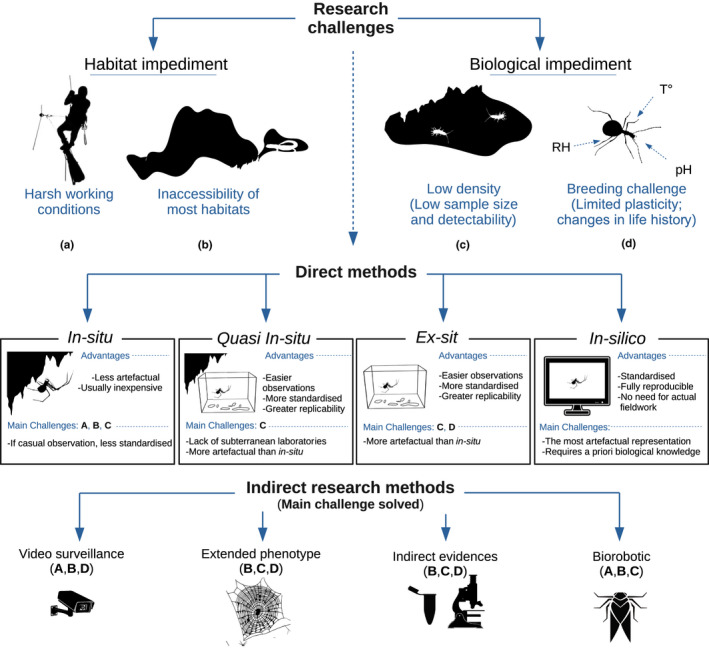
Challenges of subterranean research and experimental designs to avoid these. Schematic representation of the main challenges of subterranean research (coded with capital letters), and main experimental approaches that can be adopted to overcome these

In this work, we discuss the main impediments that we must address to standardize research in subterranean ecosystems and, subsequently, we illustrate old solutions, recommend best practices, and advance new frontiers to approach subterranean‐based studies (Figure 1). By further elaborating on the established model organisms in subterranean biology, we seek to promote caves and other subterranean habitats as experimental arenas for asking general questions in ecology, ethology, evolution, and beyond. In other words, we call for moving caves from the niche of an exotic, understudied environment to the forefront of biological science. The gain in doing so lies in the controlled conditions they offer, since they are little exposed to influences that one would have to control for in other environments.

## 2 RATIONALE FOR THIS WORK AND COMPLEMENTARY REVIEWS

It is impossible to cover all methods in subterranean biology while keeping this review tight and comprehensible. Therefore, we decided to focus on the main challenges related to subterranean research and the philosophy underlying the different experimental designs suited to overcome these: Two aspects only marginally discussed in the recent literature. Readers interested in other aspects of research in subterranean biology are referred to the classic review on biomonitoring (Culver & Sket, 2002) and published syntheses on sampling approaches (Dole‐Olivier et al., 2009; Lunghi, Corti, et al., 2020; Oliveira et al., 2019; Weinstein & Slaney, 1995; Wynne et al., 2018, 2019), species distribution modeling (Mammola & Leroy, 2018), and best practices in experimental trials with subterranean organisms (Di Lorenzo et al., 2019). Sampling techniques in non cave subterranean habitats (Box 1) have also been reviewed elsewhere—for example, boreholes (Hancock & Boulton, 2009), epikarst (Brancelj, 2004), subaquatic caves (Humphreys et al., 1999; Iliffe, 2018; Iliffe & Bowen, 2001), *Milieu Souterrain Superficiel* (Mammola et al., 2016), hyporheic (Fraser & Williams, 1997), and interstitial habitats in coastal marine and lotic environments (Schmidt‐Rhaesa, 2020).

**A modern definition of subterranean habitats and implications for research.**

The term “subterranean habitat/ecosystem” is often used as a synonym for “cave” (Mammola, 2019; Poulson & White, 1969). However, scientists have become aware that caves represent only a small fraction of the total habitat available to the subterranean fauna. More precisely, subterranean habitats comprise the breadth of underground voids of different sizes, either dry or filled with water, sharing two main ecological features: darkness and buffered climatic conditions (Culver & Pipan, 2019). These voids may open a few centimeters below ground level (Culver & Pipan, 2014) or descend several kilometers toward areas where the environmental conditions exceed the limits of life (Fišer et al., 2014). They are widespread on all continents, having been documented from different geological substrates, including carbonates (limestone and dolomite), sandstones, gypsum, granites, lava fields, iron ores, and even unconsolidated sediments (Keith et al., 2020). In summary, the cavities that we can access and explore by entering them represent just the tip of the iceberg of what lies below our feet (Ficetola et al., 2019; Mammola, Cardoso, et al., 2019).

Even though subterranean habitats are more widespread and diversified than it is usually recognized, subterranean research started with field observations in human‐accessible habitats (different types of terrestrial caves, artificial subterranean habitats such as mines and bunkers, lava tubes, cenotes, etc.), later encompassed pumped water (e.g., from drinking water wells), and only then extended to other difficult‐to‐access voids. Even today, there is still a significant research bias toward human‐accessible habitats, which should always be kept in mind. In a nutshell, it implies that we may have to relativize part of the information available to date, that is, being aware that we have mostly documented how animals behave in cave‐like environments, rather than in the extended network of fissures.

## 3 CHALLENGES TO SUBTERRANEAN RESEARCH

### 3.1 Habitat impediment

Whereas different habitats have been categorized as subterranean (Box 1), most in‐field research takes place in caves, mines, and other human‐accessible voids (Mammola, 2019). These are always dark, often muddy and humid, and sometimes even very cold, hence not offering favorable conditions to perform extensive, standardized observations (MacNeil & Brcic, 2017). There are often high ceilings, narrow fissures, and other geomorphological features that hamper the task of approaching and observing target animals without them being disturbed by light or by the presence of the researcher (CO_2_, heat, vibrations, or even diver's bubbles in the case of submerged passages). Furthermore, cave exploration requires well‐trained researchers mastering the use of speleological equipment (Zagmajster et al., 2010). Even more challenging, in this sense, are those studies set in submerged passages of freshwater and marine caves (Exley, 1983; Iliffe & Bowen, 2001), as testified by the frequent fatalities associated with cave diving (Buzzacott et al., 2009).

Given our human size, we can directly access only a small fraction of the habitats available to the subterranean fauna. As noted by Howarth (1983) (p. 380), this is a significant obstacle to scientific research because, more often than not, we cannot directly inspect the extended network of fissures “[...] *where probably the major drama in the cave ecosystem occurs*”. In a way, caves and other human‐accessible habitats may act as surrogates of the subterranean world in its entirety, windows allowing us to glimpse what usually happens away from human sight (Mammola et al., 2016; Polak, 1997; Uéno, 1987; Wilkens et al., 1986). Yet, in this case the existence of a habitat bias should be clearly acknowledged. For instance, we must be aware that the foraging behavior of a centipede that we have observed in a large chamber of a cave may not replicate in the same way—or may not even take place at all!—when the exact same centipede is dwelling in the millimetric fissures connected with the chamber. Not to mention certain typically benthic aquatic animals that have been spotted in the water column of flooded caves only after the disturbance produced by the divers (Humphreys et al., 1999).

As a corollary, however, it must be noted that a number of organisms primarily belong to human‐accessible cavities (Moseley, 2009) and *ipso facto* are more readily studied (Mammola, 2019). Classic examples are vertebrates with a centimetric body size, such as different species of cave‐roosting bats and groundwater fishes, but also the parasites and commensals associated with them (Lunghi, Ficetola, et al., 2018) or the scavengers that feed upon their carcasses and feces (Ferreira & Martins, 1999). There are also subterranean invertebrates constrained to human‐sized voids by their extended phenotypes; notably, different species of orb spiders needing larger voids for web construction (Mammola & Isaia, 2017) or aquatic suspension feeders adapted to drift in the still water column of anchialine caves (Koenemann et al., 2007; Martínez et al., 2017).

Finally, in specific subterranean systems, there may be health risks related to biological diseases or toxic gases, potentially hampering or complicating explorations and studies. Examples include fungi [*Histoplasma capsulatum* (Eurotiomycetes: Ajellomycetaceae) causing histoplasmosis; Hunt et al., 1984; Diaz, 2018; Staffolani et al., 2018], and viruses, such as Marburg virus associated with fruit bats roosting in caves (Kuzmin et al., 2010) and the potential presence of SARS‐CoV‐2 coronavirus in touristic caves (Barton, 2020). Furthermore, cave‐roosting bats may be vectors of rabies and a number of other emerging diseases (Calisher et al., 2006; Kuzmin et al., 2011)—amidst the COVID‐19 pandemic in 2020, these potential zoonotic health risks have unfortunately resulted in persecutions of bats (MacFarlane & Rocha, 2020; Rocha et al., 2020). Another human health threat may come from contaminated air due to the accumulation of hazardous gases including CO, CO_2_, and sulfur exhalations. Also, the concentration of radon (a radioactive gas) may be elevated in the subterranean realm (Cigna, 2005; Gillmore et al., 2000)—in certain caves its levels can exceed recommended doses 20‐fold.

### 3.2 Biological impediment

In several cases, the biology of subterranean species represents a further impediment to research. In general, food‐deprived subterranean environments select for long‐lived species with low metabolism and small numbers of offspring. As a consequence, the density of individuals of subterranean adapted species is often low—it is not unusual that such species were observed once at the time of their description, and never recorded thereafter (Delić & Sket, 2015; Manenti et al., 2018; Martínez et al., 2013). Also, specialized subterranean species are often unevenly distributed in space and time, mostly because they aggregate around the scarce and heterogeneously distributed food sources (Culver & Sket, 2002). These difficulties in finding sufficient individuals for experiments or in situ observations may result in studies with a reduced sample size and less robust data. This may explain why the ecology and behavior of many subterranean organisms is documented, at best, anecdotally thanks to casual observations.

Furthermore, many specialized subterranean organisms live in environments showing constant and buffered conditions and, over evolutionary time, have reduced their resilience against environmental fluctuations. For example, some terrestrial subterranean species are threatened by the smallest variations in air moisture content (Howarth, 1983), whereas aquatic animals may perish upon changes in pH driven by the water exposure to the air (Carpenter, 1999). Similarly, many terrestrial and aquatic obligate subterranean species survive only within narrow temperature ranges (Mammola, Piano, et al., 2019; Mermillod‐Blondin et al., 2013; Pallarés, Colado, et al., 2020; Pallarés, Sanchez‐Hernandez, et al., 2020). This limited physiological plasticity may pose a real challenge when a researcher is aiming to conduct experiments in the unnatural conditions of a typical laboratory. Maintaining living individuals of most of these animals is not a trivial task: breeding them requires skill and experience, in‐depth knowledge of their biology and, often, none negligible doses of luck.

In some cases, an additional impediment may be our lack of knowledge on the taxonomy of subterranean organisms (e.g., Asmyhr & Cooper, 2012; Camacho et al., 2018). This impediment (also known as 'Linnean shortfall'; Hortal et al., 2015) is especially problematic in studies focusing on community structure and functioning. It is particularly severe for tropical areas, whose subterranean fauna were largely unknown until very recently (e.g., Alvarenga et al., 2021; Trajano & Bichuette, 2010; Trajano et al., 2016), as well as for certain small‐sized animal groups which have been traditionally neglected despite being relatively abundant in subterranean habitats, such as diplurans (Sendra, Antić, et al., 2020; Sendra, Palero, et al., 2020), proturans (Galli et al., 2021), palpigrades (Mammola et al., 2021), nematodes (Du Preez et al., 2017), gastrotrichs (Kolicka et al., 2017), and other meiofaunal lineages (Martínez et al., 2019; Sánchez & Martínez, 2019). The lack of taxonomists for many groups surely hampers the conduction of more concise studies on cave communities in many regions; a situation that is further aggravated by the existence of often high cryptic diversity within most subterranean taxa (Delić et al., 2017; Eme et al., 2018; Esposito et al., 2015; Fišer et al., 2018; Gonzalez et al., 2017; Niemiller et al., 2012).

## 4 EXPERIMENTAL SETUPS

**FIGURE 2:**
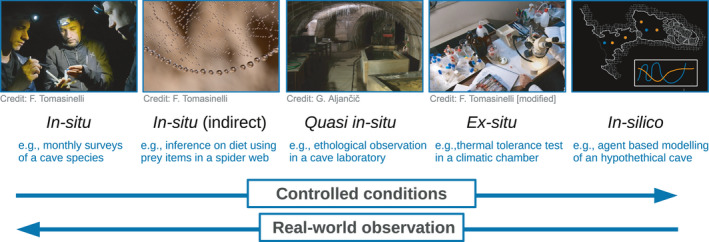
A theoretical trade‐off between the ease of study and biological realism of the observations in different experimental setups. On the one hand, exploring a cave is physically demanding and requires specific speleological equipment, whereas it is possible to run a simulation sitting at home in front of a computer in a pyjama—and even during a COVID‐19 pandemic! Running a simulation or conducting an experiment in the laboratory also allows us to control for a number of confounding factors. On the other hand, the result obtained in the field is often less artefactual, requiring no abstraction or formulation of a priori assumptions. At some point, when studying phenomena in the laboratory or with simulations, one will want to get back to the field to corroborate results using real‐world observations

The most classical and intuitive way to learn about subterranean organisms lies in quantitative observational studies, either in the field (*in situ*), under laboratory conditions (*ex situ*) or, when available, in laboratories set within caves (here termed *quasi in‐ situ*). An experimental setup entirely based on simulations (*in silico*) could also be adopted. The choice among these setups is not always straightforward. In general, choosing between alternative options is a trade‐off between the biological realism of the observations and either the ease or the extensiveness of the study (Figure 2). More detailed pros and cons of each setup are discussed in the following sections.

### 4.1 *In* *situ*

The *in* *‐* *situ* approach provides the least artefactual representation of the ecology, physiology, and behavior of the target species. Yet, this approach forces the researcher to comply with both the habitat (harsh working conditions and impossibility of exploring inaccessible habitats) and the biological (low density of most subterranean species) impediments. To minimize these impediments, a careful selection of the study site is critical. If possible, one should favor cavities with a linear development and reduced habitat complexity, thereby facilitating standardized observations (Lunghi, Corti, et al., 2020; Mammola & Isaia, 2018; Smithers, 2005) while maximizing detectability of the animals (Lunghi, 2018). In the same vein, studying aquatic target species in a semi‐submerged or shallow passageway not only increases permanence times and minimizes decompression procedures, but also maximizes safety (Iliffe, 2018).

It must be noted that *in* *‐* *situ* studies can be carried out exclusively in cavities the researcher can enter herself or at least insert instruments into. There are different types of traps and sampling devices that allow us to indirectly collect the fauna in inaccessible and interstitial habitats or even tools for detecting the presence of a species indirectly (see section “Indirect means of research”). Conversely, *in* *‐* *situ* observations are virtually impossible for porous groundwater, forcing researchers to heavily rely on laboratory studies (e.g., Di Lorenzo et al., 2014).

### 4.2 *Ex* *‐* *situ*

The use of a meso‐ or microcosmos replicating the species' natural habitat allows us to bypass the habitat impediment in its entirety. In general, obtaining standardized observations in controlled conditions enhance a greater replicability of the results, and potentially allow researchers to perform long‐lasting experiments and observations—see Carpenter (2021) for a recent example. Furthermore, *ex‐situ* approaches permit to explore the life history of those animals that prefer inaccessible habitat, or that are too small to be observed with the naked eye. Yet, by choosing an *ex‐situ* approach, the researcher needs to comply with the biological impediment of maintaining specialized and delicate organisms in the laboratory (Di Lorenzo et al., 2019), as well as with the local conservation policies for endangered species. This can be circumvented by selecting certain model organisms, often not legally protected and relatively easy to keep in the laboratory (see section “Model organisms in subterranean biology”).

As a drawback, laboratory observations may not accurately reflect the natural traits, especially behavioral and physiological, as shown in the cave (Silva et al., 2018). Although laboratory studies are useful, the *ex‐situ* conditions rarely resemble those found in the natural habitat (e.g., higher density, different environmental conditions). For well‐established model organisms, a prolonged *ex‐situ* breeding may even produce unwelcome effects such as artificial selection or adaptation to the laboratory conditions (Ross et al., 2019). This is why observations obtained from studies in the laboratory must be carefully interpreted and preferably confirmed by in situ approaches (Blin et al., 2020). For example, by surveying semi‐natural replicas of the sheltered reproductive sites of *Hydromantes* salamanders (Amphibia: Plethodontidae) with infrared cameras, Oneto et al. (2010) were able to provide some of the first observational data on their complex reproductive behavior and parental care. Subsequent observations performed under natural conditions (Lunghi, Corti, et al., 2018; Lunghi et al., 2014, 2015) confirmed the validity of these observations.

### 4.3 Quasi in‐situ

The history of subterranean biology teaches us that a *quasi in‐situ* approach—that is, to bring the laboratory into the target species’ natural habitat—eases many of the problems associated with experimental studies in the laboratory. Establishing an experimental facility within the cave itself not only spare living animals from long transportation away from the cave, but also facilitates fine‐regulation of ambient parameters within a microcosmos. The most famous example is probably the *Laboratoire Souterrain de Moulis* (*Centre national de la recherche scientifique*; CNRS), a cave‐based laboratory established in the French Pyrenees by René Jeannel (1879–1965) and Albert Vandel (1894–1980). Since its foundation in 1948, this semi‐natural experimental setting has aided generations of subterranean biologists in the challenging task of shedding light on the natural history and behavior of a wide range of elusive subterranean life forms (Clergue‐Gazeau, 1974; Durand, 1970; Juberthie, 1985; Juberthie et al., 1996; Manenti et al., 2020). For aquifers, the equivalent would be to lower sediment, substrate cages, bags into groundwater wells (Schmidt et al., 2004), which, however, would still have to be retrieved every time to study the organisms.

As a corollary, it must be noted that establishing an experimental facility inside a given cave may have a significant local impact in terms of destruction of certain microhabitats and/or alterations of the microclimate. Therefore, the establishment of similar infrastructures should be evaluated on a case‐by‐case basis and supported by an environmental risk assessment.

### 4.4 In‐silico

As a consequence of the habitat and biological impediments, studies in subterranean habitats often rely on data that is far from ideal. In a complex subterranean setting, we may lack information on environmental seasonal fluctuations, species abundances across space or time, their physiological rates and life‐history traits, or the species they interact with. Not to mention the dependency between observations and the correlation among traits (body size and trophic guild, fecundity with longevity, etc.), which often confounds with putative drivers for the process that we aim to disentangle. In those scenarios, simulations, such as agent‐based models and cellular automata, are increasingly used to explore the dynamics of natural ecosystems and trigger novel ideas for further exploration in real‐world settings (DeAngelis & Grimm, 2014). These mechanistic models rely on the so‐called “first principles,” such as energy budgets, physiology, or fitness seeking (Grimm & Berger, 2016), which define the initial conditions of the simulation so that behavior and interactions emerge rather than being imposed by the modeler. Given robust enough assumptions, simulations are thus able to realistically replicate sets of empirical patterns without restricting them to a single deterministic scenario (Grimm et al., 2005). For example, the use of eco‐evolutionary agent‐based models, which include heritable traits and the use of genetic algorithms, provides insights on the evolution of certain morphological, physiological, and behavioral traits (Ayllón et al., 2018).

Surprisingly, however, simulations have rarely been applied in subterranean biology. Applications to subsurface systems so far have been restricted to porous groundwater, with the focus being mainly on contaminant degradation (Benioug et al., 2015, 2017; Schmidt et al., 2018; Tang et al., 2013), and to soils (e.g., Banitz et al., 2013; Borer et al., 2019; Kim & Or, 2016). It is easy to see how the simulation of a virtual cave would be an interesting aid to research. Caves may represent ideal model systems for *in‐silico* studies due to their constant environmental conditions, which can be easily and predictably simulated, and their simple community structure with few species and limited interactions. For example, these models would allow us to achieve a mechanistic understanding of the processes behind interactions between species within a typical subterranean community, to explore pathways of subterranean evolution, and even to elucidate the impact of climate change on subterranean biodiversity.

The applicability of these theoretical models to the real biological world, however, still depends on the quality and availability of data. Parametrization of simulations might be relatively simple for broad questions in spatial or temporal scope, but quite complex for very specific systems, often implying the need for possessing detailed information. Thus, and this is true for other methods as well, the necessity to parametrize theoretical models with the real‐world biological observations may require combining simulation approaches with actual fieldwork. Importantly, models may single out those parameters that warrant the most attention and may thus steer experiments toward focussing on sensitive and critical parameters. A complementary avenue is combining qualitative observations, for example, that state changes are confined within a certain interval, for parameterization. Even if a single observation does not contain much information, a combination of several qualitative observations can be as distinctive as a single high‐precision observation. This inverse, “pattern‐oriented” parameterization (Grimm et al., 2005; Wiegand et al., 2004), has been proven to be a powerful approach and overlaps with the more formal approximate Bayesian computing approach (Hartig et al., 2011).

### 4.5 Indirect means of research

A plethora of indirect methodologies can be used to overcome both the habitat and the biological impediments (Figure 1). These approaches are mostly species‐ and system‐specific, and it is impossible to provide widely general recommendations. Therefore, we here discuss examples chosen to illustrate the concept of “indirect research.”

Information about the ecology and behavior of large‐sized animals can be acquired via infrared video surveillance. This represents a low‐cost and low‐personnel effort methodology, which has a long tradition in ethological research and biomonitoring (Swann et al., 2004). In caves, thermal‐infrared imaging and laser scanning have been extensively applied to study the swarming and roosting behaviors of bats (Azmy et al., 2012; Elliott et al., 2005), but could potentially be used for other vertebrates, such as cave salamanders (Lunghi, Manenti, et al., 2020). In at least one case, camera trapping has even been used to quantifying wildlife use of cave entrances (Baker, 2015).

Recently, there has also been a great deal of discussion on the use of molecular tools to obtain indirect evidence of the presence and behavior of species, especially in difficult‐to‐access habitats, as well as to overcome prevalent taxonomic biases (Malard et al., 2020). For example, environmental DNA was successfully used to detect the presence of focal subterranean species, such as amphibians (Gorički et al., 2017) and crustaceans (Boyd et al., 2020; DiStefano et al., 2020; Niemiller et al., 2018). The analysis of gut or stomach content of species inhabiting both human‐accessible and interstitial environments provides information on dietary requirements and trophic behaviors taking place in both these compartments (Lunghi, Cianferoni, et al., 2018; Lunghi, Manenti, et al., 2020), but also trophic web studies with aquatic subterranean species (Saccò et al., 2019). These analyses can be done visually, but also through massive sequencing techniques, allowing the identification of the gut content using DNA (Rastorgueff et al., 2015). Similarly, stable isotopes proved useful to understand species interactions and niche partitioning (Chávez‐Solís et al., 2020), as well as identifying potential carbon sources through space (Brankovits et al., 2017) and time (Saccò et al., 2020).

In some circumstances, the species' extended phenotype also informs indirectly on specific behaviors and ecological needs. The web in web‐building spiders, for example, can be viewed as an extended phenotype that enlarges the sensory world of its builder in interaction with the environment (Blamires, 2010). The web also provides a record frozen in time of the spider's foraging behavior, as spiders modify their webs in response to a large range of biotic and abiotic stimuli, including previous prey experiences, climatic variables, and the structural complexity of the habitat (Hesselberg, 2015; Vollrath & Selden, 2007). The easily quantifiable two‐dimensional orb‐web, in particular, is highly suitable for behavioral studies, as orb spiders can easily be maintained in the laboratory (Zschokke & Herberstein, 2005) or their webs measured in the field (Hesselberg, 2010). The ubiquity of orb‐web spiders near the entrance of temperate caves makes this approach especially promising (Hesselberg et al., 2019). Likewise, the calcified tubes of several hard‐bodied aquatic organisms, such as tube‐building polychaetes, bring us information on the evolution of aquatic caves communities and paleoclimate from past geological eras (Moldovan et al., 2011).

The living world has long been used as a source for developing biologically inspired robots using biomimetics design principles to provide innovative technical solutions (Lenau et al., 2018; Pfeifer et al., 2007; Vincent et al., 2006). In recent years, the use of biorobotic models to test and generate biological hypotheses has been gaining ground (Gravish & Lauder, 2018). Following this recent trend, we propose that the use of small, agile biorobots to explore, record, and interact with subterranean animals in their natural habitats might overcome many of the habitat and biological impediments previously discussed (Woodward & Sitti, 2014). For example, the use of a simple biomimetic robot fish has been successfully used to highlight similarities and differences in social behavior between surface and cave‐dwelling populations of *Poecilia mexicana* (Actinopterygii: Poeciliidae) (Bierbach et al., 2018).

## 5 MODEL ORGANISMS IN SUBTERRANEAN BIOLOGY

Model organisms represent only a small part of Earth's biodiversity and yet have largely contributed to our knowledge on many fields within the biological sciences (Hedges, 2002). The earliest models, such as flies, mice, or roundworms, were selected for the task simply because they were small, proliferative, and easy to culture and manipulate; they were, however, quite limiting in advancing many aspects in ecology and evolution. Luckily, the growth of modern molecular methods, staining and imaging techniques, and gene editing, have facilitated choosing more appropriate models for the biological question at hand rather than enforcing the ones that can be easily grown and manipulated (Müller & Grossniklaus, 2010). Consequently, the number of model species has diversified along with the number scientific questions, and now includes representatives of many animal phyla (as well as plants and fungi). This exciting transition in contemporary biology is embodied by the term “non‐model” organism, which reflects that the diversity of model species has grown nearly parallel with the diversity of problems addressed (Goldstein & King, 2016; Russell et al., 2017; Sullivan, 2015).

**FIGURE 3:**
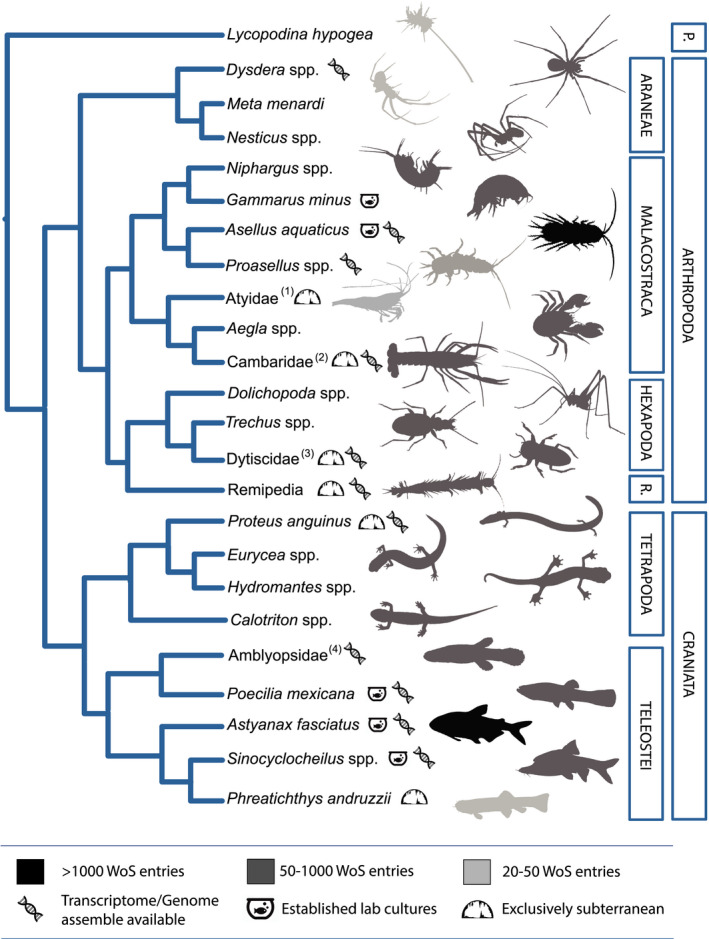
Diversity of model organisms in subterranean biology across the animal Tree of Life. The branch Cambaridae refers to the genera *Cambarus*, *Orconectes*, *Procambarus*, and *Troglocambarus*. Atyidae refers to the exclusively subterranean genera *Speleocaris*, *Stygiocaris*, *Troglocaris*, and *Typhlatya*. Dytiscidae indicates the Australian diving beetles of the genera *Limbodesus*, *Nirridesus*, *Nirripirti*, and *Paroster*. Amblyopsidae indicates the North American cave fish in the genera *Amblyopsis*, *Chologaster*, *Forbesichthys*, *Speleoplatyrhinus*, and *Typhichthys*. WoS entries: Number of papers focusing on the species in Web of Science (accessed on 25 November 2020). (1–3): The information refers to the genera (1) *Speleocaris*, *Stygiocaris*, *Troglocaris*, and *Typhlatya*; (2) *Cambarus*, *Orconectes*, *Procambarus*, and *Troglocambarus*; (3) *Paroster*, *Limbodesus*, *Nirridesus*, and *Nirripirti*

The trend of diversification of model systems and research question is evident in cave biology as well. To comprehend it, we have compiled a list of those subterranean animals that can be considered as model organisms (Table S1). We selected models based on two criteria: (a) organisms/groups with accumulated at least 20 papers in the Web of Science (accessed on 25 November 2020); and (b) organisms/groups with at least two independent research laboratories focusing on them. Our list of model organisms includes species with different degrees of subterranean specialization across three phyla, but it is dominated by Teleostei fish and Crustacea (Figure 3). This reflects the traditional research bias in subterranean biology toward these groups, only partially justified by their dominance across subterranean environments. Only a few of these species satisfy the traditional requirement of a model—successful culturing in the lab and keeping long‐standing laboratory breeds [e.g., *Astyanax mexicanus* (Actinopterygii: Characidae), *Asellus aquaticus* (Isopoda: Asellidae), and *Poecillia mexicana*]. By far, the most famous and studied among these is the cavefish *Astyanax mexicanus* (Jeffery, 2020; Keene et al., 2016; Torres‐Paz et al., 2018), which has been kept in captivity for many generations (Wilkens, 1971) and is increasingly used and recognized as suitable for tackling problems beyond the typical subterranean biology realm (Maher, 2009; McGaugh et al., 2020). Other models thrive in laboratory conditions, but are unable to complete their life cycle therein [e.g., *Gammarus minus* (Amphipoda: Gammaridae), Australian calcrete Dytiscidae insects]. Most models in subterranean biology are lineages with both surface and subterranean populations, or species whose populations exhibit different degrees of subterranean specialization. Among those, *Astyanax mexicanus* and *Asellus aquaticus* are even able to form hybrid offspring between cave and surface morphs in laboratory conditions (Jeffery, 2020; Protas & Jeffery, 2012).

Alongside every other biological discipline, cave biology research has entered the genomics era (Friedrich, 2013; Pérez‐Moreno et al., 2016). Already half of cave models listed in Table S1 have been included in genome (transcriptome) sequencing projects, becoming windows into the molecular basis of adaptation (Barbosa et al., 2017; Berning et al., 2019). With the decreasing prices and the development of more user friendly bioinformatic recourses, so‐called ‐*omics* tools will soon be at the forefront of cave research and exploited in the remaining model systems. Such tools may enable overcoming traditional restrictions on the use of subterranean species as models and we predict that the peculiar, and even bizarre, traits of subterranean animals are going to draw attention from an increasingly wider audience, and possibly attract new researchers into the field (Mammola et al., 2020).

In subterranean biology, the concept of model organism has also been applied to supra‐specific lineages widely used to investigate evolutionary processes associated with cave colonization or to answer biogeographic and macroecological questions. Similar studies typically rely on comparative methods within explicit phylogenetic frameworks, allowing us to distinguish the role played by ecological adaptations and evolutionary history on the observed ecological and distribution patterns (Juan et al., 2010; Mammola et al., 2020). Some of these models account for lineages including both surface and subterranean species exhibiting different degrees of adaptations and ecological preferences, such as *Asellus* (Verovnik et al., 2004), *Niphargus* (Amphipoda: Niphargidae) (Fišer, 2009)*, Trechus* (Coleoptera: Carabidae) (Möst et al., 2020), and *Dysdera* (Araneae: Dysderidae) (Arnedo et al., 2007). Others exclusively consist of subterranean species, such as atyd shrimps (Decapoda: Atyidae) of the genera *Typhlatya*, *Stygiocaris*, *Speleocaris*, and *Troglocaris* (Jurado‐Rivera et al., 2017; Zakšek et al., 2009). While lineages in the first group are useful to understand different mechanisms for ecological speciation and habitat shift, subterranean‐exclusive lineages allow us to understand the role of historical stochastic processes in subterranean diversity and biogeography (Juan et al., 2010). In addition, subterranean‐exclusive lineages have been studied in comparison with distantly surface‐dwelling relatives to understand the adaptation processes related to the colonization of the subterranean environment [e.g., *Phreatichthys andruzzii* (Actinopterygii: Cyprinidae)]. Although intuitively less ideal, this approach has yielded some important insights, such as the impacts of life in darkness on the circadian clock or DNA repair mechanisms (Cavallari et al., 2011).

Finally, some subterranean species with unique features have been established as models to investigate scientific questions not necessarily related to the classic subterranean research agenda. This is the case of the carnivorous sponge *Lycopodina hypogea* (Demospongiae: Cladorhizidae), used as a model for early nervous system evolution and developmental biology (Godefroy et al., 2019); the crustaceans in the class Remipedia, key to understanding the evolution of terrestrial arthropods (Lozano‐Fernandez et al., 2016), the evolution of the nervous system (Stemme et al., 2013), and venoms toxins (von Reumont et al., 2014); or the “forever young” aquatic salamander *Proteus anguinus* (Amphibia: Proteidae), whose progenetic origin and long lifespan has triggered fruitful research on the molecular mechanisms of aging (Voituron et al., 2011). Also, *A. mexicanus* have been increasingly used as a model organism in various areas of biomedical research, such as diabetes (Riddle et al., 2018), insomnia (Jaggard et al., 2018), autism (Yoshizawa et al., 2018), and regeneration (Stockdale et al., 2018). While those are not the questions that have inspired most cave‐based researchers over the years, they have recently attracted considerable interest and funding, thereby illustrating the general idea of our review here: Caves, in their uniqueness for humans, still hold the secrets for understanding broad scientific questions (Martínez & Mammola, 2020).

## 6 CONCLUSIONS

In this work, we discussed best practices and novel ideas for performing standardized research in subterranean ecosystems, by focusing on key impediments, experimental ideas, and model systems. The main take‐home messages that emerge from this exercise are as follows:

1. *Be aware of the many options out there*. Insofar as each subterranean system and organism is unique to some extent, and in light of the impediments to subterranean research, scientists must be creative in designing their experiments. Research in subterranean biology often implies combining traditional in situ field observations with standardized studies in a laboratory setting, either within a cave (*quasi in‐situ*) or outside the cave (*ex‐situ*). It is also important to be aware of the potential of novel tools, especially simulations, artificial intelligence methods, and biorobotics (Figure 1).
2. *Choose the right model*. Many impediments to subterranean research can be overcome by focusing on model organisms, which have been established owing to their specific traits and/or their broad availability in subterranean environments. Whereas model systems in subterranean biology are probably not as developed as in other disciplines, there are options across the animal tree of life offering great potential for tackling specific research questions (Figure 3). Since a major challenge before fully exploiting a given model is to breed it in the laboratory, it would be worthwhile endeavor to run a wider screening among candidate organisms. In this way, a model suitable to answer a given set of questions and able to complete its lifecycle in the laboratory can be identified.
3. *Be aware of the taxonomic bias*. As a corollary of the previous point, it is important to remember that our knowledge of subterranean species is still strongly biased in its taxonomical coverage. Even today, the natural history information on subterranean species remains largely fragmented, rarely standardized, and often biased toward a few well‐studied model organisms and temperate regions. We stress the importance of broadening eco‐evolutionary studies to incorporate a larger range of organisms and subterranean habitats, to explore hypotheses about the emergence of convergent traits and behaviors across distant taxa while accounting for phylogenetic effects.
4. *Embrace multidisciplinarity*. In light of the habitat and biological impediments, combining ecological and behavioral observations with evolutionary approaches, genetic tools, and simulations are a critical premise. In the ‐*omics* era, integrative studies are expected to grow, allowing us to understand which molecular adjustments (including epigenetic effects) occur during the surface‐subterranean transitions. This is required, for example, to disentangle the role of standing genetic variation and phenotypic plasticity in driving the evolution of subterranean populations (Bilandžija et al., 2020).

## CONFLICTS OF INTEREST

None declared.

## AUTHOR CONTRIBUTIONS

SM wrote the first draft. AM and SIS provided most arguments on aquatic habitats. EL and HB provided most arguments on vertebrates. AM, SM, and HB developed the section on model organisms. PC, SIS, and VG developed the in silico section. TH provided expert opinion on different sections, especially on behavioral topics. AM and SM prepared figures. All authors contributed to the writing with comments and additions.

## Supporting information

Table S1

## Data Availability

This review contains no data. Number of papers and molecular sequences included in Table S1 have been derived from Web of Science and GeneBank (accessed on 5 January 2021).
