# Supplementary material for: Collecting eco‐evolutionary data in the dark: Impediments to subterranean research and how to overcome them
Source: Ecol Evol. 2021 May 1;11(11):5911–26. doi: 10.1002/ece3.7556 (PMC8207145; doi:10.1002/ece3.7556)
Supplement: Supplementary file 1 — Table S1 [file ECE3-11-5911-s001.pdf]

Table S1. Example of model organisms in subterranean biology. Model were selected based on two criteria: i) Organisms/groups with at least 20 papers listed in the Web Of Science; ii) Organisms/groups with at least two research labs focusing on them. Lab = Is the species reared in laboratory conditions and kept in stable cultures?; Habitat = Aq.: aquatic; Terr.: terrestrial; Lab = is the species commonly reared in the lab? Gen = Is a genome/transcriptome for the species available?; Level = Population: research on the species mainly based on comparing different populations; Species: research on the organism mainly based on comparison of different species; No. Species = Number of species in the genus/group. WoS = Number of paper in Web of Science; GeneBank = Number of sequence available in GeneBank; Ref = reference in which the species is suggested as a model system; * the organism have not been yet referred a as model in the literature.

Higher taxonomy	Species/Group	Ecology	Habitat	Lab	Gen	Level	Examples of applications	No. species	WoS	Gene Bank	Ref
Porifera	*Lycopodina hypogea (=A. hypogea)*	Subterranean exclusive	Aq.	no	no	Population	Nervous system evolution, developmental biology (Adamska, 2016; Borchiellini et al. 2020); micro-eukarya interactions (Dupont et al. 2013; 2014); trophic ecology (Rastorgueff et al. 2011)	1	20	150	Godefroy et al. 2019
Malacostraca	*Gammarus minus*	Surface/ Subterranean	Aq.	yes	yes	Population	Subterranean adaptation (Carlini et al. 2017; MacAvoy et al. 2016), ecotoxicology (Chapman 2017), microbiome (Moriarty 2017), phylogeography (Carlini et al. 2009)	1	82	670	Fong 2019
	Niphargus spp.	Surface/ Subterranean	Aq.	no	no	Species	Subterranean adaptation (Trontelj et al. 2009; Copilas-Ciocianu et al. 2018), phylogeny and phylogeography (Fišer et al. 2012, Perez-Moreno et al. 2017), niche-based studies (Fišer et al. 2012), ecotoxicology (Avramov et al. 2013)	411, mosty subterranean	200	5517	Fišer 2019
	Asellus aquaticus	Surface/ Subterranean	Aq.	yes	yes	Population	Subterranean adaptation (Protas et al. 2011; Stahl et al. 2015), phylogeography (Sworobowicz et al. 2015), ecotoxicology (Bloor 2010)	1	609	22864	Protas & Jeffery 2012; Verovnik & Konec 2019
	Proasellus spp.	Surface/ Subterranean	Aq.	no	yes	Species	Trophic ecology (Francois et al. 2016), physiology (Mermillod-Blondin et al. 2013), ecotoxicology (Mösslacher 2000), phylogeography (Eme et al. 2013), community ecology (Weitowitz et al. 2019)	76	67	3148857	-
	Aegla spp.	Surface/ Subterranean	Aq.	no	no	Population/ Species	Phylogeography (Loretán et al. 2020), morphological adaptation (Fernandes & Bichuette, 2013, Marchiori et al. 2015)	90	201	3195	Cogo et al. 2018
	Cambaridae (*Cambarus, Procambarus, Orconectes, Troglocambarus*)*	Surface/ Subterranean	Aq.	no	yes	Species	Phylogeography (Buhay et al. 2007), subterranean adaptation (Mejia Ortiz & Hartnoll, 2005; Cooper et al. 2001) molecular mechanisms (Stern & Crandall. 2018), behavior (Li & Cooper, 2002), ecotoxicology (Mathews et al. 1977), invasive species (Mouser et al. 2019)	ca. 40 cave species out of ca. 200	124*	1177	-
	Atyidae (*Typhlatya, Troglocaris, Spelaeocaris, Stygiocaris*)*	Subterranean exclusive	Aq.	no	no	Species	Phylogeography (Jurado-Rivera et al. 2017, Zakšek et al. 2009), trophic ecology (Chávez-Solís et al. 2020), adaptation (Jugović et al. 2010, Bilandžija et al. 2017)	19	21	794	-
Remipedia	Remipedia*	Subterranean exclusive	Aq.	no	yes	Species	Arthopod evolution (Lozano-Fernandez et al. 2016), venom (von Reumont et al. 2014), nervous system (Fanenbruck et al. 2004, Stemme et al. 2013), phylogeography (Neiber et al. 2011)	30	105	109807	-

Hexapoda	*Dolichopoda* spp. *	Surface/ Subterranean	Terr.	no	no	Population/ Species	Subterranean adaptation (Sbordoni et al. 2000; Strauß & Sritih, 2017), ecotoxicology (Augustyniak et al. 2015)	ca. 50	72	1134	-
	Trechus spp.*	Surface/ Subterranean	Terr.	no	no	Population/ Species	Global change biology (Brandmayer et al. 2013, Rizzo et al. 2015), phylogeography (Contreras-Diaz et al. 2007, Faille et al 2013, Möst et al. 2020)	> 1000	127	1780	-
	Australian calcrete Dytiscidae (*Paroster, Limbodesus, Nirridesus, Nirripirti*)	Surface/ Subterranean	Aq.	no	yes	Species	Phylogeny and phylogeography (Cooper et al. 2002, Guzik et al. 2009), morphology (Alarie et al. 2009), trophic ecology (Sacco et al. 2020), subterranean adaptation (Jones et al. 2019), molecular mechanisms (Tierney et al. 2015), behavior (Langille et al. 2019)	>100 cave adapted	73*	1831*	Bilton et al. 2019.
Arachnida	*Meta menardi* (and related species)	Surface/ Subterranean	Terr.	no	no	Population/ Species	Subterranean adaptation (Hesselberg et al. 2019; Lipovšek et al. 2018), niche-based studied (Novak et al. 2010; Mammola & Isaia 2014, 2017), behaviour (Hesselberg et al. 2019), trophic ecology (Novak et al. 2010), silk studies (Lepore et al. 2012)	3	34	31	Hesselberg et al. 2019; Mammola et al. 2020
	Dysdera spp.	Surface/ Subterranean	Terr.	no	yes	Species	Subterranean adaptation (Arnedo et al. 2007), phylogeography (Macias-Hernandez et al. 2008), trophic ecology (Vizueta et al. 2019)	286	72	1928	Arnedo et al. 2007
	Nesticus spp. *	Surface/ Subterranean	Terr.	no	no	Species	Subterranean adaptation (Sanchez-Herrero et al. 2019), cryptic species, behaviour (Huber, 1993), phylogeography (Hedin 1997; Snowman et al. 2010)	279	54	323	-
Teleostei	*Astyanax mexicanus*	Surface/ Subterranean	Aq.	yes	yes	Population	Subterranean adaptation and mechanisms (Wilkens 1988; Jeffery 2009; Bilandžija et al. 2020), evolutionary history (Bradić et al., 2012; Gross 2012), nervous system (Rétaux et al. 2013), behaviour (Yoshizawa et al. 2010; Dubue et al. 2011), medicine (Riddle et al. 2018, Stockdale et al. 2018)	1	1426	251767	Wilkens, 2010; Protas & Jeffery 2012
	Amblyopsidae (*Amblyopsis, Chologaster, Forbesichthys, Speoplatyrhinus, Typhlichthys*)	Surface/ Subterranean	Aq.	no	yes	Species	Subterranean adaptations (Poulson, 1963, Niemiller et al 2013a, Niemiller et al 2013b), phylogeny (Hart et al. 2020)	8	95	1509	Niemiller & Poulson, 2010
	*Phreatichthys andruzzii**	Subterranean exclusive	Aq.	yes	no	Species	Behavior (Paglianti et al. 2006, Tartellin et al, 2011), subterranean adaptation (Berti et al. 2001), molecular mechanisms (Zhao et al. 2018, Stemmer et al. 2015, Ceinos et al. 2018)	1	33	62	-
	Sinocyclocheilus spp.	Surface/ Subterranean	Aq.	yes	yes	Population/ Species	Subterranean adaptation (Xiao et al. 2005), nervous system (Meng et al. 2018), molecular mechanisms adptation (Meng et al. 2013)	67	76	813399	Yang et al. 2016
	Poecilia mexicana	Surface/ Subterranean	Aq.	yes	yes	Population	Subterranean adaptation phylogeography (Plath et al. 2007), nervous system (McGowan et al. 2019), molecular mechanisms adptation (Passow et al. 2017)	1	205	79924	Kelley et al. 2012

Tetrapoda	*Hydromantes* spp.* (= *Speleomantes* spp.)	Surface/ Subterranean	Terr.	no	no	Species	Subterranean adaptation (Adams et al. 2019), nervous system (Dicke et al. 1997), behaviour, trophic ecology (Lunghi et al. 2018)	8	121	2255	-
	Eurycea spp.*	Surface/ Subterranean	Aq./ Terr.	no	no	Species	Phylogeography (Edgington et al 2016) subterranean adaptations (Phillips et al. 2017, Bendik et al. 2013; Edgington & Taylor, 2019), trophic ecology (Fenolio et al 2006), parasitology (McAlister & Bursey 2004)	ca. 30	414	3629	-
	Proteus anguinus	Subterranean exclusive	Aq.	no	yes	-	Subterranean adaptation, behaviour (Hervant et al. 2001, Schlegel et al 2009; Balázs et al. 2020), ageing and progenesis (Voituron et al. 2011), parasitology (Omar et al. 2019)	1	112	198	Aljančič et al. 2019
	Calotriton spp. (=*Euproctus* spp.)	Surface/ Subterranean	Aq./ Terr.	no	yes	Population/ Species	Trophic ecology (Manenti et al. 2020); phylogeography (Caccone et al. 1997, Rovelli et al. 2019), thermal biology (Trochet 2018), behaviour (Hervant et al. 2001)	3	121	894	-

Literature cited

Adams DC & Nistri A (2010). Ontogenetic convergence and evolution of foot morphology in European cave salamanders (Family: Plethodontidae). BMC Evolutionary Biology 10.1: 216.

Adamska M (2016). Sponges as models to study emergence of complex animals. Current Opinion in Genetics & Development, 39, 21-28.

Alarie Y, Michat MC & Watts CH (2009). Larval morphology of Paroster Sharp, 1882 (Coleoptera: Dytiscidae: Hydroporinae): reinforcement of the hypothesis of monophyletic origin and discussion of phenotypic accommodation to a hypogaeic environment. Zootaxa, 2274(1), 1-44.

Aljančič G (2019). History of research on Proteus anguinus Laurenti 1768 in Slovenia/Zgodovina raziskovanja človeške ribice (Proteus anguinus Laurenti 1768) v Sloveniji. Folia biologica et geologica, 60(1), 39-69.

Augustyniak M, Gladysz M & Dziewięcka M (2016) The Comet assay in insects—Status, prospects and benefits for science. Mutation Research/Reviews in Mutation Research 767: 67-76.

Arnedo MA, Oromí P, Múrria C, Macías-Hernández N, Ribera C 2007 The dark side of an island radiation: systematics and evolution of troglobitic spiders of the genus Dysdera Latreille (Araneae : Dysderidae) in the Canary Islands. Invert. Syst. 21, 623.

Avramov M, Schmidt SI & Griebler C (2013). A new bioassay for the ecotoxicological testing of VOCs on groundwater invertebrates and the effects of toluene on Niphargus inopinatus. Aquatic toxicology, 130, 1-8.

Balázs G, Lewarne B & Herczeg G (2020). Extreme site fidelity of the olm (Proteus anguinus) revealed by a long-term capture–mark–recapture study. Journal of Zoolog

Bendik NF, Morrison TA, Gluesenkamp, AG, Sanders MS & O'Donnell LJ (2013). Computer-assisted photo identification outperforms visible implant elastomers in an endangered salamander, Eurycea tonkawaE PloS one, 8(3), e59424.

Berti R, Durand JP, Becchi S, Brizzi R, Keller N, & Ruffat G (2001). Eye degeneration in the blind cave-dwelling fish Phreatichthys andruzzii. Canadian journal of zoology, 79(7), 1278-1285.

Bilandžija H, Laslo M, Porter ML & Fong DW (2017). Melanization in response to wounding is ancestral in arthropods and conserved in albino cave species. Scientific reports, 7(1), 1-11.

Bilandžija H, Hollifield B, Steck M, Meng G, Ng M, Koch AD, Gračan R, Ćetković H, Porter ML & Jeffery W (2020). Phenotypic plasticity as a mechanism of cave colonization and adaptation. Elife, 9, e51830.

Bilton DT, Ribera I & Short AEZ (2019). Water beetles as models in ecology and evolution. Annual Review of Entomology 64: 359-377.

Bloor M (2010). Animal standardisation for mixed species ecotoxicological studies: establishing a laboratory breeding programme for Gammarus pulex and Asellus aquaticus. Zoologica baetica 21: 179-190.

Borchiellini C, Degnan SM, Le Goff E, Rocher C, Vernale A, Baghdiguian S, Séjourné N, Marschal F, Le Bivic A, Godefroy N, Degnan BM & Renard E (2020) Staining and Tracking Methods for Studying Sponge Cell Dynamics. In Developmental Biology of the Sea Urchin and Other Marine Invertebrates (pp. 81-97). Humana, New York, NY.

Bradic M, Beerli P, García-de León FJ, Esquivel-Bobadilla S & Borowsky RL. (2012) Gene flow and population structure in the Mexican blind cavefish complex (Astyanax mexicanus). BMC Evol Biol (12)9.

Brandmayr P, Giorgi F, Casale A, Colombetta G, Mariotti L, Vigna Taglianti A, Weber F & Pizzolotto R (2013). Hypogean carabid beetles as indicators of global warming?. Environmental Research Letters, 8(4), 044047.

Buhay JE, Moni G, Mann N & Crandall KA (2007). Molecular taxonomy in the dark: evolutionary history, phylogeography, and diversity of cave crayfish in the subgenus Aviticambarus, genus Cambarus. Molecular Phylogenetics and Evolution, 42(2), 435-448.

Caccone A, Milinkovitch MC, Sbordoni V, Powell JR (1997). Mitochondrial DNA rates and biogeography in European newts (genus Euproctus). Systematic biology, 46(1), 126-144.

Carlini DB, Manning J, Sullivan PG & Fong DW. 2009. Molecular genetic variation and population structure in morphologically differentiated cave and surface populations of the freshwater amphipod Gammarus minus. Mol Ecol 18:1932–1945. doi:10.1111/j.1365-294X.2009.04161.x

Carlini DB & Fong DW (2017) The transcriptomes of cave and surface populations of Gammarus minus (Crustacea: Amphipoda) provide evidence for positive selection on cave downregulated transcripts. PLoS One 12.10: e0186173.

Ceinos RM, Frigato E, Pagano C, Fröhlich N, Negrini P, Cavallari N, Vallone D, Fuselli S, Bertolucci C & Foulkes NS (2018). Mutations in blind cavefish target the light-regulated circadian clock gene, period 2. Scientific reports, 8(1), 1-11.

Chapman T (2017) Enemy Exacerbation: Effects of Predator Stress on Sulfate Lethality in Freshwater Amphipods (Gammarus minus). Electronic Theses and Dissertations. Paper 3297. https://dc.etsu.edu/etd/3297

Chávez-Solís, E M, Solís C, Simões N, & Mascaró M (2020). Distribution patterns, carbon sources and niche partitioning in cave shrimps (Atyidae: Typhlatya). Scientific reports, 10(1), 1-16.

Cogo, GB, Biasi C, Severo ES, Loro V, & Santos S (2018). Environmental warming induces behavioral and metabolic changes in a freshwater crustacean− aeglids as a model organism. In Annales de Limnologie-International Journal of Limnology (Vol. 54, p. 7). EDP Sciences.

Contreras-Díaz, H G, Moya O, Oromí P, & Juan, C (2007). Evolution and diversification of the forest and hypogean ground-beetle genus Trechus in the Canary Islands. Molecular Phylogenetics and Evolution, 42(3), 687-699.

Cooper SJB, Hinze S, Leys R, Watts CHS, & Humphreys, W. F (2002). Islands under the desert: molecular systematics and evolutionary origins of stygobitic water beetles (Coleoptera: Dytiscidae) from central Western AustraliAInvertebrate systematics, 16(4), 589-590.

Cooper RL, Li H, Long LY, Cole JL & Hopper HL (2001). Anatomical comparisons of neural systems in sighted epigean and troglobitic crayfish species. Journal of Crustacean Biology, 21(2), 360-374.

Copilaş-Ciocianu D, Fišer C, Borza P & Petrusek A (2018). Is subterranean lifestyle reversible? Independent and recent large-scale dispersal into surface waters by two species of the groundwater amphipod genus Niphargus. Molecular Phylogenetics and Evolution, 119, 37-49.

Dicke U, Wallstein M & Roth G (1997). 5-HT-like immunoreactivity in the brains of plethodontid and salamandrid salamanders (Hydromantes italicus, Hydromantes genei, Plethodon jordani, Desmognathus ochrophaeus, Pleurodeles waltl): an immunohistochemical and biocytin double-labelling study. Cell and tissue research 287.3: 513-523.

Duboué ER, Keene AC, Borowsky RL. (2011) Evolutionary convergence on sleep loss in cavefish populations. Curr Biol (21) 671–676.

Dupont S, Carre-Mlouka A, Li YY, Vacelet J & Bourguet-Kondracki, ML (2013, October). Diversity of bacteria associated with the Carnivorous Sponge Asbestopluma hypogea. In CYTOTECHNOLOGY (Vol. 65, No. 5, pp. 686-687).

Dupont S, Carre-Mlouka A, Domart-Coulon I, Vacelet J & Bourguet-Kondracki ML (2014). Exploring cultivable Bacteria from the prokaryotic community associated with the carnivorous sponge Asbestopluma hypogea. FEMS microbiology ecology, 88(1), 160-174.

Edgington HA & Taylor DR (2019). Ecological contributions to body shape evolution in salamanders of the genus Eurycea (Plethodontidae). PLoS ONE 14(5): e0216754

Edgington HA, Ingram CM & Taylor DR (2016). Cyto-nuclear discordance suggests complex evolutionary history in the cave-dwelling salamander, Eurycea lucifuga. Ecology and Evolution 6(17): 6121-6138.

Eme D, Malard F, Konecny−Dupré L, Lefébure T & Douady CJ. (2013). Bayesian phylogeographic inferences reveal contrasting colonization dynamics among E uropean groundwater isopods. Molecular Ecology, 22(22), 5685-5699.

Faille A, Casale A, Balke M & Ribera I (2013). A molecular phylogeny of Alpine subterranean Trechini (Coleoptera: Carabidae). BMC Evolutionary Biology, 13(1), 1-16.

Fanenbruck M, Harzsch S & Wägele JW (2004) The brain of the Remipedia (Crustacea) and an alternative hypothesis on their phylogenetic relationships. Proceedings of the National Academy of Sciences 101.11: 3868-3873.

Fenolio DB, Graening GO, Collier BA & Stout JF (2006). Coprophagy in a cave-adapted salamander; the importance of bat guano examined through nutritional and stable isotope analyses. Proceedings of the Royal Society B: Biological Sciences, 273(1585), 439-443.

Fernandes C & Bichuette M (2013) Shape variation of Aegla schmitti (Crustacea, Decapoda, Aeglidae) associated to superficial and subterranean stream reaches." Subterranean Biology 10: 17

Fišer C, Blejec A & Trontelj P (2012). Niche-based mechanisms operating within extreme habitats: a case study of subterranean amphipod communities. Biology Letters, 8(4), 578-581.

Fišer C, Sket B & Trontelj P (2008). A phylogenetic perspective on 160 years of troubled taxonomy of Niphargus (Crustacea: Amphipoda). Zoologica Scripta, (37) 665-680.

Fišer C (2019). Niphargus—A model system for evolution and ecology. In Encyclopedia of caves (pp. 746-755). Academic Press.

Fong DW (2019). Gammarus minus: a model system for the study of adaptation to the cave environment. In Encyclopedia of caves (pp. 451-458). Academic Press.

Francois CM, Mermillod−Blondin F, Malard F, Fourel F, Lécuyer C, Douady CJ & Simon, L (2016). Trophic ecology of groundwater species reveals specialization in a low−productivity environment. Functional ecology, 30(2), 262-273.

Gamage AM, Zhu F, Ahn M, Foo RJH, Hey YY, Low DH, Mendenhall IH, Dutertre CA & Wang LF (2020). Immunophenotyping monocytes, macrophages and granulocytes in the Pteropodid bat Eonycteris spelaea. Scientific reports, 10(1), 1-16.

García-París M & Deban SM (1995) A novel antipredator mechanism in salamanders: rolling escape in Hydromantes platycephalus. Journal of herpetology 29.1: 149-151.

Godefroy N, Le Goff E, Martinand-Mari C, Belkhir K, Vacelet J & Baghdiguian, S (2019). Sponge digestive system diversity and evolution: filter feeding to carnivory. Cell and tissue research, 1-11.

Gross JB. (2012) The complex origin of Astyanax cavefish. BMC Evol Biol (12)105.

Guzik MT, Cooper SJB, Humphreys WF & Austin AD (2009). Fine–scale comparative phylogeography of a sympatric sister species triplet of subterranean diving beetles from a single calcrete aquifer in Western AustraliAMolecular Ecology, 18(17), 3683-3698.

Hart PB, Niemiller ML, Burress ED, Armbruster JW, Ludt WB & Chakrabarty P (2020). Cave–adapted evolution in the North American amblyopsid fishes inferred using phylogenomics and geometric morphometrics. Evolution.

Hedin MC(1997). Molecular phylogenetics at the population/species interface in cave spiders of the Southern Appalachians (Araneae: Nesticidae: Nesticus). Molecular Biology and Evolution, 14(3), 309-324.

Hesselberg T, Simonsen D & Juan C (2019). Do cave orb spiders show unique behavioural adaptations to subterranean life? A review of the evidence. Behaviour, 1–28.

Hervant F, Mathieu J & Durand J (2001). Behavioural, physiological and metabolic responses to long-term starvation and refeeding in a blind cave-dwelling (Proteus anguinus) and a surface-dwelling (Euproctus asper) salamander. Journal of Experimental Biology, 204(2), 269-281.

Huber BA(1993). Genital mechanics and sexual selection in the spider Nesticus cellulanus (Araneae: Nesticidae). Canadian journal of zoology, 71(12), 2437-2447.

Jeffery WR. (2009). Regressive Evolution in Astyanax Cavefish. Annu Rev Genet 43, 25–47.

Jones KK, Cooper SJ & Seymour RS (2019). Cutaneous respiration by diving beetles from underground aquifers of Western Australia (Coleoptera: Dytiscidae). Journal of Experimental Biology, 222(7), jeb196659.

Jurado-Rivera JA, Pons J, Alvarez F, Botello A, Humphreys WF, Page TJ, Iliffe TM, Willasen E, Meland K, Juan C & Jaume D (2017). Phylogenetic evidence that both ancient vicariance and dispersal have contributed to the biogeographic patterns of anchialine cave shrimps. Scientific reports, 7(1), 1-11.

Jugovic J, Prevorcnik S, Aljancic G & Sket B (2010). The atyid shrimp (Crustacea: Decapoda: Atyidae) rostrum: phylogeny versus adaptation, taxonomy versus trophic ecology, Journal of Natural History, 44, 41-42, 2509-2533.

Kelley JL, Passow, CN, Plath M, Rodriguez LA, Yee MC & Tobler M (2012). Genomic resources for a model in adaptation and speciation research: characterization of the Poecilia mexicana transcriptomE BMC genomics, 13(1), 652.

Langille BL, Tierney SM, Austin AD, Humphreys WF & Cooper SJ (2019). How blind are they? Phototactic responses in stygobiont diving beetles (Coleoptera: Dytiscidae) from calcrete aquifers of Western AustraliAAustral Entomology, 58(2), 425-431.

Lepore E, Marchioro A, Isaia M, Buehler MJ & Pugno NM (2012). Evidence of the Most Stretchable Egg Sac Silk Stalk, of the European Spider of the Year Meta menardi. PLOS ONE, 7(2), e30500.

Li H & Cooper RL (2002). The effect of ambient light on blind cave crayfish: social interactions. Journal of Crustacean Biology, 22(2), 449-458.

Lipovšek S, Novak T, Janžekovič F, Brdelak N & Leitinger G (2018). Changes in the midgut diverticula epithelial cells of the European cave spider, Meta menardi, under controlled winter starvation. Scientific reports, 8(1), 1-13.

Loretán G, Rueda EC, Cabrera JM, Pérez-Losada M, Collins PA & Giri F (2020). Geographical isolation and restricted gene flow drive speciation of Aegla singularis (Decapoda: Anomura: Aeglidae) in southern South AmericABiological Journal of the Linnean Society, 129(1), 177-189.

Lozano-Fernandez J, Carton R, Tanner AR, Puttick MN, Blaxter M, Vinther J, Olensen J, Giribet G, Edgecombe, GD & Pisani, D (2016). A molecular palaeobiological exploration of arthropod terrestrialization. Philosophical Transactions of the Royal Society B: Biological Sciences, 371(1699), 20150133.

Lunghi E, Cianferoni F, Ceccolini F, Veith M, Manenti R, Mancinelli G, Corti C, & Ficetola GF (2018). What shapes the trophic niche of European plethodontid salamanders?. PloS one, 13(10), e0205672.

MacAvoy SE, Braciszewski A, Tengi E & Fong DW (2016). Trophic plasticity among spring vs. cave populations of Gammarus minus: examining functional niches using stable isotopes and C/N ratios. Ecological research, 31(4), 589-595.

Macias-Hernandez N, Oromí P & Arnedo MA (2008) Patterns of diversification on old volcanic islands as revealed by the woodlouse-hunter spider genus Dysdera (Araneae, Dysderidae) in the eastern Canary Islands. Biological Journal of the Linnean Society 94.3: 589-615.

Mammola S & Isaia M (2014). Niche differentiation in Meta bourneti and M menardi (Araneae, Tetragnathidae) with notes on the life history. International Journal of Speleology, 43 (3), 343- 353.

Mammola S & Isaia M (2017) Rapid poleward distributional shifts in the European cave–dwelling Meta spiders under the influence of competition dynamics. Journal of Biogeography 44: 2789-2797.

Mammola S, Hesselberg T, Lunghi E (2020) A trade-off between latitude and elevation contributes to explain range segregation of broadly distributed cave-dwelling spiders. Journal of Zoological Systematics and Evolutionary Research, doi: 10.1111/jzs.12432

Manenti R, Melotto A, Guillaume O, Ficetola GF & Lunghi E (2020). Switching from mesopredator to apex predator: how do responses vary in amphibians adapted to cave living?. Behavioral Ecology and Sociobiology, 74(10), 1-13.

Marchiori AB, Fornel R, Santos S (2015) Morphometric variation in allopatric populations of Aegla platensis (Crustacea: Decapoda: Anomura): possible evidence for cryptic speciation. Zoomorphology 134.1: 45-53.

Mathews RC, Bosnak AD, Tennant DS & Morgan EL (1977). Mortality curves of blind cave crayfish (Orconectes australis australis) exposed to chlorinated stream water. Hydrobiologia, 53(2), 107-111.

McAllister CT & Bursey CR (2004). Endoparasites of the dark-sided salamander, Eurycea longicauda melanopleura, and the cave salamander, Eurycea lucifuga (Caudata: Plethodontidae), from two caves in Arkansas, USAComparative Parasitology, 71(1), 61-66.

McGowan KL, Passow CN, Arias-Rodriguez L, Tobler M & Kelley JL (2019). Expression analyses of cave mollies (Poecilia mexicana) reveal key genes involved in the early evolution of eye regression. Biology letters, 15(10), 20190554.

Mejía-Ortíz LM & Hartnoll RG (2005). Modifications of eye structure and integumental pigment in two cave crayfish. Journal of Crustacean Biology, 25(3), 480-487.

Meng F, Braasch I, Phillips JB, Lin X, Titus T, Zhang C & Postlethwait JH (2013). Evolution of the eye transcriptome under constant darkness in Sinocyclocheilus cavefish. Molecular biology and evolution, 30(7), 1527-1543.

Meng F, Zhao Y, Titus T, Zhang C & Postlethwait JH (2018). Brain of the blind: transcriptomics of the golden-line cavefish brain. Current Zoology, 64(6), 765-773.

Mermillod-Blondin F, Lefour C, Lalouette L, Renault D, Malard F, Simon L & Douady CJ (2013). Thermal tolerance breadths among groundwater crustaceans living in a thermally constant environment. Journal of Experimental Biology, 216(9), 1683-1694.

Moriarty, J. Investigating the Coevoluation of the Microbiome of Surface and Cave Populations of the Amphipod Crustacean Gammarus minus. Diss. American University, 2017.

Mösslacher F (2000). Sensitivity of groundwater and surface water crustaceans to chemical pollutants and hypoxia: implications for pollution management. Archiv für Hydrobiologie, 51-66.

Möst MH, Donabauer M, Arthofer W, Schlick-Steiner BC & Steiner FM (2020). Towards an evolutionary history of European-Alpine Trechus ground beetles: species groups and wing reduction. Molecular Phylogenetics and Evolution, 149, 106822.

Mouser J, Ashley DC, Aley T & Brewer SK (2019). Subterranean invasion by gapped ringed crayfish: Effectiveness of a removal effort and barrier installation. Diversity, 11(1), 3.

Neiber MT, Hartke TR, Stemme T, Bergmann A, Rust J, Iliffe TM & Koenemann S (2011). Global biodiversity and phylogenetic evaluation of Remipedia (Crustacea). PLoS One, 6(5), e19627.

Niemiller ML, Graening GO, Fenolio DB, Godwin JC, Cooley JR, Pearson WD, Fitzpatrick BM & Near TJ (2013). Doomed before they are described? The need for conservation assessments of cryptic species complexes using an amblyopsid cavefish (Amblyopsidae: Typhlichthys) as a case study. Biodiversity and Conservation, 22(8), 1799-1820.

Niemiller ML, Fitzpatrick BM, Shah P, Schmitz L & Near TJ (2013). Evidence for repeated loss of selective constraint in rhodopsin of amblyopsid cavefishes (Teleostei: Amblyopsidae). Evolution: International Journal of Organic Evolution, 67(3), 732-748.

Niemiller ML & Poulson TL (2010). Subterranean fishes of North America: Amblyopsidae. Biology of subterranean fishes, 169, 280.

Novak T, Tkvac T, Kuntner M, Arnett EA, Delakorda SL, Perc M & Janžekovič F (2010). Niche partitioning in orbweaving spider Meta menardi and Metellina merianae (Tetragnathidae). Acta Oecologica, 36: 522-529.

Omar MA, Heckmann AR, Žiga F, Zakšek V, Holger H, Kostanjšek R. (2019) Description of Acanthocephalus anguillae balkanicus subsp. n. (Acanthocephala: Echinorhynchidae) from Proteus anguinus Laurenti (Amphibia: Proteidae) and the cave ecomorph of Asellus aquaticus (Crustacea: Asellidae) in Slovenia. Folia Parasitologica 66, 1-13.

Paglianti A, Messana G, Cianfanelli A & Berti R (2006). Is the perception of their own odour effective in orienting the exploratory activity of cave fishes?. Canadian journal of zoology, 84(6), 871-876.

Passow CN, Brown AP, Arias‐Rodriguez L, Yee MC, Sockell A, Schartl M, Warren WC, Bustamante C, Kelley JL & Tobler M (2017). Complexities of gene expression patterns in natural populations of an extremophile fish (Poecilia mexicana, Poeciliidae). Molecular ecology, 26(16), 4211-4225.

Pérez-Moreno JL, Balázs G, Wilkins B, Herczeg G & Bracken-Grissom HD (2017). The role of isolation on contrasting phylogeographic patterns in two cave crustaceans. BMC evolutionary biology, 17(1), 247.

Phillips JG, Fenolio DB, Emel SL & Bonett RM (2017). Hydrologic and geologic history of the Ozark Plateau drive phylogenomic patterns in a cave‐obligate salamander. Journal of Biogeography, 44(11), 2463-2474.

Plath M, Hauswaldt JS, Moll K, Tobler M, García de León FJ, Schlupp I & Tiedemann R (2007). Local adaptation and pronounced genetic differentiation in an extremophile fish, Poecilia mexicana, inhabiting a Mexican cave with toxic hydrogen sulphide. Molecular Ecology, 16(5), 967-976.

Poulson TL (1963). Cave adaptation in amblyopsid fishes. American Midland Naturalist, 257-290.

Protas, ME, Trontelj P & Patel NH (2011) Genetic basis of eye and pigment loss in the cave crustacean, Asellus aquaticus. Proceedings of the National Academy of Sciences 108.14: 5702-5707.

Protas M & Jeffery WR (2012). Evolution and development in cave animals: from fish to crustaceans. Wiley Interdisciplinary Reviews: Developmental Biology, 1(6), 823-845.

Rastorgueff PA, Harmelin-Vivien M, Richard P & Chevaldonné P (2011). Feeding strategies and resource partitioning mitigate the effects of oligotrophy for marine cave mysids. Marine Ecology Progress Series, 440, 163-176.

Rétaux S, Alié A, Blin M, Devos L, Elipot Y & Hinaux H (2016). Neural development and evolution in Astyanax mexicanus: comparing cavefish and surface fish brains. Biology and evolution of the Mexican cavefish, 223-240.

Riddle MR, Aspiras AC, Gaudenz K, Peuß R, Sung JY, Martineau B, Peavey M, Box AC, Tabin JA, McGaugh S, Borowsky R, Tabin CJ & Rohner N (2018). Insulin resistance in cavefish as an adaptation to a nutrient-limited environment. Nature, 555(7698), 647-651.

Rizzo V, Sánchez-Fernández D, Fresneda J, Cieslak A, & Ribera I (2015). Lack of evolutionary adjustment to ambient temperature in highly specialized cave beetles. BMC evolutionary biology, 15(1), 10.

Rovelli V, Ruiz-González A, Vignoli L, Macale D, Buono, V, Davoli F, Vietes DR, Pezaro N & Randi E (2019). Genotyping-by-Sequencing (GBS) of large amphibian genomes: a comparative study of two non-model species endemic to Italy. Animal Biology, 69(3), 307-326.

Saccò M, Blyth AJ, Humphreys WF, Karasiewicz S, Meredith KT, Laini A, Cooper SJB, Bateman PW & Grice K (2020). Stygofaunal community trends along varied rainfall conditions: deciphering ecological niche dynamics of a shallow calcrete in Western Australia. Ecohydrology, 13(1), e2150.

Sánchez-Herrero JF, Frías-López C, Escuer P, Hinojosa-Alvarez S, Arnedo MA, Sánchez-Gracia A & Rozas J (2019). The draft genome sequence of the spider Dysdera silvatica (Araneae, Dysderidae): A valuable resource for functional and evolutionary genomic studies in chelicerates. GigaScience, 8(8), giz099.

Sbordoni V, Allegrucci G & Cesaroni D (2000). Population genetic structure, speciation and evolutionary rates in cave dwelling organisms. Subterranean Ecosystems (Ecosystems of the World 30) (pp. 453-477). Amsterdam : Elsevier SciencE

Schlegel PA, Steinfartz S, Bulog B (2009). Non-visual sensory physiology and magnetic orientation in the Blind Cave Salamander, Proteus anguinus (and some other cave-dwelling urodele species). Review and new results on light-sensitivity and non-visual orientation in subterranean urodeles (Amphibia). Animal biology, 59(3), 351-384.

Snowman CV, Zigler KS & Hedin M (2010). Caves as islands: mitochondrial phylogeography of the cave-obligate spider species Nesticus barri (Araneae: Nesticidae). The Journal of Arachnology, 38(1), 49-56.

Stahl BA, Gross JB, Speiser DI, Oakley TH, Patel NH, Gould DB & Protas ME (2015). A transcriptomic analysis of cave, surface, and hybrid isopod crustaceans of the species Asellus aquaticus. PloS one, 10(10), e0140484.

Stemme T, Iliffe TM, von Reumont BM, Koenemann S, Harzsch S & Bicker G (2013). Serotonin-immunoreactive neurons in the ventral nerve cord of Remipedia (Crustacea): support for a sister group relationship of Remipedia and Hexapoda?. BMC evolutionary biology, 13(1), 1-15.

Stemmer M, Schuhmacher LN, Foulkes NS, Bertolucci C & Wittbrodt J (2015). Cavefish eye loss in response to an early block in retinal differentiation progression. Development, 142(4), 743-752.

Stern DB & Crandall KA(2018). Phototransduction gene expression and evolution in cave and surface crayfishes. Integrative and comparative biology, 58(3), 398-410.

Strauß J & Stritih N (2017). Neuronal regression of internal leg vibroreceptor organs in a cave-dwelling insect (Orthoptera: Rhaphidophoridae: Dolichopoda araneiformis). Brain, behavior and evolution 89.2: 104-116.

Stockdale WT, Lemieux ME, Killen AC, Zhao J, Hu Z, Riepsaame J, Hamilton N, Kudoh T, Riley PR, van Aerle R, Yamamoto Y, Mommersteeg MTM. (2018) Heart Regeneration in the Mexican Cavefish. Cell Rep (25) 1997-2007.

Sworobowicz L, Grabowski M, Mamos T, Burzyński A, Kilikowska A, Sell J & Wysocka A (2015). Revisiting the phylogeography of Asellus aquaticus in Europe: insights into cryptic diversity and spatiotemporal diversification. Freshwater Biology, 60(9), 1824-1840.

Tarttelin EE, Frigato E, Bellingham J, Di Rosa V, Berti R, Foulkes NS, Lucas RJ & Bertolucci C (2012). Encephalic photoreception and phototactic response in the troglobiont Somalian blind cavefish Phreatichthys andruzzii. Journal of Experimental Biology, 215(16), 2898-2903.

Tierney SM, Cooper SJ, Saint KM, Bertozzi T, Hyde J, Humphreys WF & Austin AD (2015). Opsin transcripts of predatory diving beetles: a comparison of surface and subterranean photic niches. Royal Society open science, 2(1), 140386.

Trochet A, Dupoué A, Souchet J, Bertrand R, Deluen M., Murarasu S, Calvez O, Martiínez-Silvestre A, Verdaguer-Foz I, Darnet E, Le Chevalier H, Mossoll M, Guillaume O, Aubret F (2018). Variation of preferred body temperatures along an altitudinal gradient: A multi-species study. Journal of thermal biology, 77, 38-44.

Trontelj P, Douady CJ, Fišer C, Gibert J, Gorički Š, Lefebure T, Sket, B & Zakšek V (2009). A molecular test for cryptic diversity in ground water: how large are the ranges of macro−stygobionts?. Freshwater Biology, 54(4), 727-744.

Verovnik R & Konec M (2019). Asellus aquaticus: A model system for historical biogeography. In Encyclopedia of caves (pp. 76-84). Academic Press.

Vizueta J, Macías−Hernández N, Arnedo MA, Rozas J & Sánchez−Gracia A (2019). Chance and predictability in evolution: the genomic basis of convergent dietary specializations in an adaptive radiation. Molecular ecology, 28(17), 4028-4045.

Voituron Y, de Fraipont M, Issartel J, Guillaume O & Clobert J (2011). Extreme lifespan of the human fish (Proteus anguinus): a challenge for ageing mechanisms. Biology Letters, 7(1), 105-107.

von Reumont BM, Blanke A, Richter S, Alvarez F, Bleidorn C & Jenner RA(2014). The first venomous crustacean revealed by transcriptomics and functional morphology: remipede venom glands express a unique toxin cocktail dominated by enzymes and a neurotoxin. Molecular biology and evolution, 31(1), 48-58.

Weitowitz DC, Robertson AL, Bloomfield JP, Maurice L & Reiss J (2019). Obligate groundwater crustaceans mediate biofilm interactions in a subsurface food weBFreshwater Science, 38(3), 491-502.

Wen M, Ng JH, Zhu F, Chionh YT, Chia WN, Mendenhall, I. H, ... & Wang, L F (2018). Exploring the genome and transcriptome of the cave nectar bat Eonycteris spelaea with PacBio long-read sequencing. GigaScience, 7(10), giy116.

Wilkens H. (1988) Evolution and genetics of epigean and cave Astyanax fasciatus (Characidae, Pisces). Evol Biol (23) 271–367.

Wilkens, H (2010). Genes, modules and the evolution of cave fish. Heredity 105.5: 413-422.

Xiao H, Chen SY, Liu ZM, Zhang RD, Li WX, Zan RG & Zhang YP (2005). Molecular phylogeny of Sinocyclocheilus (Cypriniformes: Cyprinidae) inferred from mitochondrial DNA sequences. Molecular Phylogenetics and Evolution, 36(1), 67-77.

Yang J, Chen X, Bai J, Fang D, Qiu Y, Jiang, W., ... & Pan, X (2016). The Sinocyclocheilus cavefish genome provides insights into cave adaptation. BMC biology, 14(1), 1-13.

Yoshizawa M, Gorički Š, Soares D, Jeffery WR. (2010) Evolution of a behavioral shift mediated by superficial neuromasts helps cavefish find food in darkness. Curr Biol (20) 1631–1636.

Zhao H, Di Mauro G, Lungu-Mitea S, Negrini P, Guarino AM, Frigato E, Braunbeck T, Ma H, Lamparter T, Vallone C, Bertolucci C & Foulkes NS (2018). Modulation of DNA repair systems in blind cavefish during evolution in constant darkness. Current Biology, 28(20), 3229-3243.

Zakšek V, Sket B, Gottstein S, Franjević D & Trontelj P (2009). The limits of cryptic diversity in groundwater: phylogeography of the cave shrimp Troglocaris anophthalmus (Crustacea: Decapoda: Atyidae). Molecular Ecology, 18(5), 931-946.
